# Eyestrain (Commonly Called Asthenopia)

**Published:** 1893-06

**Authors:** 


*Eyestrain (commonly called Asthenopia).* By ERNEST CLARKE, M.D. Pp. viii., 168. London: J. & A. CHURCHILL. 1892.

This small handbook will be found of value to all practitioners who may be called upon to treat cases of frontal neuralgia or headache, as well as to ophthalmic surgeons. Much of what has been written on the symptoms of eyestrain is referred to and elaborated. Various forms of asthenopia—accommodative, muscular, and retinal—are described and explained. Refractive errors, neuroses, and muscular insufficiencies and anomalies are fully considered, together with their treatment by lenses, prisms, or operation. The index to the book and the references to authors show that the writer has not taken any narrow view of his subject.

